# Disrupted Circadian Rhythms and Substance Use Disorders: A Narrative Review

**DOI:** 10.3390/clockssleep6030030

**Published:** 2024-08-19

**Authors:** Pallavi Sharma, Randy J. Nelson

**Affiliations:** Department of Neuroscience, Rockefeller Neuroscience Institute, West Virginia University, Morgantown, WV 26506, USA; randy.nelson@hsc.wvu.edu

**Keywords:** artificial light at night, circadian rhythms, clock genes, sleep, substance use disorders

## Abstract

Substance use disorder is a major global health concern, with a high prevalence among adolescents and young adults. The most common substances of abuse include alcohol, marijuana, cocaine, nicotine, and opiates. Evidence suggests that a mismatch between contemporary lifestyle and environmental demands leads to disrupted circadian rhythms that impair optimal physiological and behavioral function, which can increase the vulnerability to develop substance use disorder and related problems. The circadian system plays an important role in regulating the sleep–wake cycle and reward processing, both of which directly affect substance abuse. Distorted substance use can have a reciprocal effect on the circadian system by influencing circadian clock gene expression. Considering the detrimental health consequences and profound societal impact of substance use disorder, it is crucial to comprehend its complex association with circadian rhythms, which can pave the way for the generation of novel chronotherapeutic treatment approaches. In this narrative review, we have explored the potential contributions of disrupted circadian rhythms and sleep on use and relapse of different substances of abuse. The involvement of circadian clock genes with drug reward pathways is discussed, along with the potential research areas that can be explored to minimize disordered substance use by improving circadian hygiene.

## 1. Introduction

Several natural rhythms are observed on Earth, including the tides, the seasons of the year, and the day-night cycles. In response to the rotation of the Earth around its axis, virtually all life forms have developed an internal temporal representation of the solar day. These so-called circadian rhythms are endogenous cycles generated by central and peripheral biological circadian clocks [1,2]. The circadian clocks maintain optimal biochemical, molecular, physiological, and behavioral rhythms, including the sleep–wake cycle, metabolism, body temperature, gene expression, and hormone secretion. 

To synchronize (entrain) these internal rhythms precisely to the 24 h daily environmental light–dark cycles, animals rely on external rhythmic time cues or *Zeitgebers*. Light is the strongest entraining cue for humans [3]. 

The eyes play an essential role in light detection and regulating various image-forming, as well as nonimage-forming visual functions [4]. In addition to activating the visual photoreceptors involved in vision, light is also detected by the intrinsically photosensitive retinal ganglion cells (ipRGCs) in the eyes [5,6,7]. The photopigment activated by light in the ipRGCs is melanopsin [6,7], which is maximally sensitive to short wavelength (blue) light, with λ_max_ at approximately 480 nm (peak sensitivity from 447 to 484 nm) [8,9]. The spectrum sensitivity of human melanopsin is comparable to that of rodents, supporting the role of these lab animals in studies related to the alteration of this system with light or pharmaceuticals [10]. 

The circadian system is hierarchically organized [11]. Light information is conveyed via the retinohypothalamic tract to the suprachiasmatic nuclei (SCN) also known as the central circadian clock. Neural and humoral signals from the SCN synchronize various central and peripheral oscillators, resulting in integrated temporal coordination for optimal function [12,13]. The circadian clock is conserved phylogenetically from cyanobacteria to humans and functions as a transcriptional–translational feedback loop [14]. 

Modern lifestyle factors such as artificial lighting, uneven sleep–wake routine, lengthy work hours, and distorted eating habits can impair circadian entrainment by disturbing the natural light–dark cycle that synchronizes the circadian clocks [15,16,17]. Disruption of circadian rhythms can result in initiating or aggravating several health problems, such as inflammatory responses, metabolic disorders, affective disorders, as well as chronic issues such as cancer, diabetes, cognitive deficits, cardiovascular, and neuropsychological disorders [18,19]. Moreover, a growing body of evidence suggests that disruption of circadian rhythms can affect sleep and reward mechanisms enhancing the susceptibility to misuse of substances [20,21,22]. The role of the circadian system in substance use disorders can be observed at the molecular and genetic level. The circadian system affects the bioenergetic pathways in dopamine neurons that drive drug reward and addiction [23]. Also, circadian clock genes can influence the midbrain dopaminergic activity [24]. Genetic aberrations in circadian mechanisms can predispose a person to disordered substance use, while prolonged circadian disruptions, as experienced by shift workers and adolescents, may enhance vulnerability to substance use disorders [21,25]. On the other hand, misuse of drugs and other substances can directly cause sleep disturbances and alter the expression of circadian clock genes, which can increase the probability for relapse to substance abuse [22]. 

**Figure 1:**
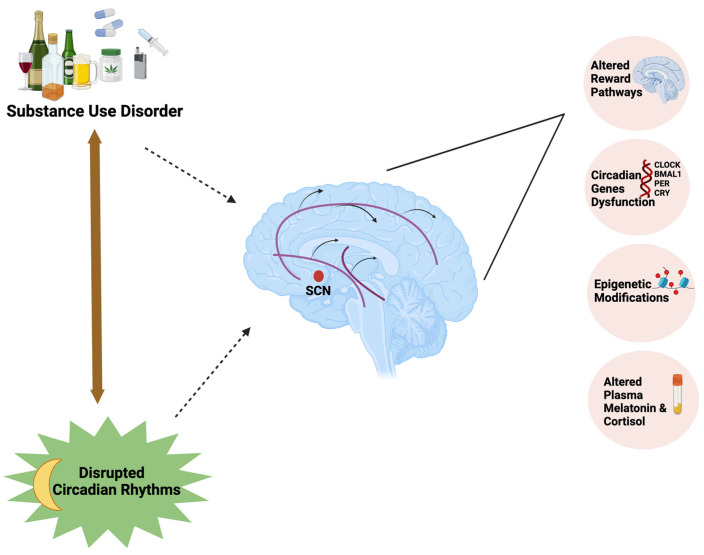
Substance use disorder forms a bidirectional relationship with circadian rhythm disruption by influencing the mesolimbic dopaminergic pathways, expression of circadian clock genes, and epigenetic modifications and affecting plasma melatonin and cortisol concentrations.

In the current review, we explored different factors influencing circadian rhythms and the underlying mechanisms linking the circadian rhythm disruption with substance use disorders (Figure 1). The literature search was conducted using Google Scholar and PubMed with relevant search terms including: Circadian rhythms, Substance abuse, Substance use disorder, Clock genes, Dopaminergic pathways, Chronotype, Alcohol use disorder, Opioids, Effect of circadian rhythm disruption on substance use, Effect of artificial light on circadian system, Substance use disorder in adolescents, and follow-up searches as warranted.

## 2. Factors Influencing Circadian Rhythms

In order to maintain optimal mental and physical health, it is important to synchronize the sleep–wake/rest–activity cycles with the environmental light–dark cycles [26]. Disrupted circadian rhythms can be caused by a number of factors such as night shift work, social jetlag, psychiatric issues, and use of certain drugs [27]. Indoor artificial light exposure during the day is often too dim to entrain human circadian rhythms [28]. In contrast, exposure to artificial light at night (ALAN) can interfere with synchronization of the circadian rhythms because the spectral content is typically blue-enriched [29]. Below, we present major factors that can lead to circadian rhythm disruption and hence potentially contribute to substance abuse. 

### 2.1. Sleep Disturbances

The sleep–wake cycle is likely the most salient of our circadian rhythms with sleep timing and duration as the most critical aspects [30]. Sleep is regulated by two processes: the circadian clock system and the sleep homeostat [31]. The circadian system and sleep are interrelated by various metabolic and genetic factors [32,33]. Therefore, disturbances in either of them can have similar cognitive, metabolic, and immunological consequences [32,34]. 

Because circadian rhythm disruptors also impair sleep, it is difficult to determine whether the negative downstream effects are due to disrupted circadian rhythms, disrupted sleep, or both. One, but not the only, approach to separate out the effects of disrupted circadian rhythms versus disrupted sleep is to study nocturnal mice that sleep during the day when there is light [35]. Many people are susceptible to environmental factors that disrupt circadian rhythms and cause sleep disturbances, whereas some individuals are genetically prone to circadian misalignment and sleep-related issues [36,37]. 

Inadequate and improperly scheduled sleep can interfere with the expression and transcription of circadian clock genes in human and rodents by disturbing the coherence of the multi-oscillator system comprising core and peripheral oscillators. This further interrupts the molecular pathways related to metabolism, hormone signaling, immune function, inflammation, cell cycle, and stress responses [38]. The misalignment between the endogenous circadian rhythms and sleep–wake cycle can result in circadian rhythm sleep disorder, characterized by a recurring pattern of sleep disruption resulting in insomnia, excessive daytime sleepiness, psychological problems, and functional deficits that decline the overall quality of life [39,40]. 

### 2.2. Eveningness and Morningness Chronotypes

People may align differently to the light–dark cycle due to genetic diversity and environmental influences that affect the endogenous biological clock [41]. “Chronotype” is a person’s innate tendency for sleep and wake activities, influenced by circadian rhythms [42,43]. Generally, chronotype reflects a distribution of individuals between the two extremes: (1) morning types, who prefer to sleep and wake up early (colloquial morning larks), and (2) evening types, who prefer to sleep and wake up later (night owls) [2,43]. Inter-individual variance in daily alignment of activities is attributed to how individual clocks respond to light and dark [2]. Various studies have been performed on the behavioral patterns among eveningness and morningness chronotypes. Adolescents who do not have sufficient sleep are more likely to develop obesity and mood disorders [44,45,46]. An inclination towards the eveningness chronotype, along with insomnia and mood disorders, can be correlated with an increased risk of substance-related disorders [47,48].

### 2.3. Social Jetlag

The term social jetlag was first introduced in 2006 [49]. Social jetlag is the temporal misalignment in the timing of the mid-point of sleep during nights before school or employment and those before work-free days [18]. Because most people live according to social demands (work or school schedules), their chronotype (and social jetlag) is evident on work-free days. Late chronotypes generally show large disparities in sleep–wake schedules. An estimated 70% of working professionals and students experience at least one hour of social jetlag, with nearly 50% suffering more than two hours [18]. Approximately 80% of people with daily work schedules were reported to use alarm clocks on workdays [3,50]. Most adolescents wake up before their natural wake-up time on weekdays and stay awake longer than usual on weekends [51]. People often try to counterbalance this sleep debt accumulated during the working days by oversleeping on their free days [49]. Social jetlag is reported to be associated with an increased risk of obesity [50], diabetes [52], prostate cancer [53], as well as endocrine and cardiovascular issues [54]. Social jetlag and insufficient sleep were reported to produce more depressive symptoms in adolescent females than in males [55].

### 2.4. Night Shift Work

Night shift workers often experience substantial social jetlag. Night shift workers are reported to experience more sleep disturbances than non-shift workers [56]. Because of their frequently phase-shifting exposure or rest–activity patterns, night shift workers display symptoms similar to those of jetlag [57]. Many of the long-term negative effects of night shift work on health may even become apparent after employees have stopped working night shifts [58]. 

Night shift workers typically sleep during the day and are awake much of the night. However, when traveling to and from work or during their personal time, they are exposed to the natural day and night light conditions. This leads to a clash between the contrasting lighting conditions (i.e., natural during the commute and artificial at work) [59]. Because of this desynchronization, night shift workers often experience issues such as altered melatonin and cortisol secretions, insomnia, excessive daytime sleepiness, and fatigue which impede their overall performance [60,61]. 

### 2.5. Use of Electronic Devices at Night 

The use of electronic devices at night, especially those with light-emitting screens, has long been related to disruptions to sleep and circadian rhythms. Nighttime texting and the use of electronic devices before bedtime are significantly associated with delayed and shorter sleep times in adolescents [62,63]. Exposure to blue light and electromagnetic fields from mobile phones can change melatonin onset time, promote rapid eye movement (REM) sleep, and alter the sleep electroencephalogram [64,65]. In a study performed during the corona virus disease (COVID-19) lockdown, participants with increased electronic device usage show decreased sleep quality, prolonged sleep onset latency, reduced sleep duration, and exacerbated insomnia symptoms along with delayed bedtime and rising times [66]. In another study investigating the effect of light wavelengths and intensity, it was observed that light wavelength has a greater impact on sleep and other biological functions than light intensity [67]. In a study comparing the effect of evening exposure of blue (460 nm) and green light (550 nm), it was observed that blue light exposure can change sleep EEG power spectra by reducing slow-wave activity during the first sleep cycle and decreasing REM sleep duration [68]. Another study confirmed that young adults with delayed sleep timing possess a greater intrinsic ipRGC responsiveness to blue light [69]. Students restricting their screen time after 21:00 (9 PM) reported earlier sleep onset time, longer overall sleep duration, and increased daytime vigilance [70]. 

### 2.6. Disruption of Circadian Rhythms in Healthcare Facilities 

Patients in health care facilities frequently experience disruption of sleep and circadian rhythms. For example, nursing home residents commonly experience impaired nighttime sleep, as well as increased daytime sleeping and diminished expression of circadian rhythms [71]. Unusual lighting, perturbed food intake schedules, noise, prolonged patient interactions, and medication can all contribute to disturbed circadian rhythms [72]. Problems with sleep onset, frequent awakening, and poor-quality sleep are commonly reported in hospitalized patients. All of these factors likely impede patients’ healing and increase their length of stay [73]. A randomized crossover trial confirmed that after residing in a nursing home with blue light-depleted light sources, healthy adults displayed augmented melatonin concentrations coupled with melatonin phase-advancement, improved sleep quality, and decreased neurocognitive arousal [74]. Thus, in order to maximize patient care and outcomes, it is crucial to address the circadian rhythm-related issues in healthcare facilities. 

### 2.7. Sex-Related Differences in Circadian Rhythms

Studies reveal significant differences in circadian rhythms between males and females. In comparison to men, women experience lower body temperature amplitudes and higher melatonin secretion amplitudes. Also, women have earlier circadian phases of core body temperature and pineal melatonin secretion relative to sleep time [75]. Sex-related disparities are observed in the circadian rhythmicity of cognitive functioning as well, with women showing more nighttime impairment in cognitive performance than men [76]. Generally, women have shorter intrinsic circadian periods than men. A large proportion of women have circadian periods shorter than 24 h, suggestive of women’s predilection for morning activities and earlier waking hours [77]. It was observed that the majority of target sites that receive direct SCN input express gonadal steroid receptors [78]. Therefore, gonadal hormones may play a crucial role in controlling the response of the circadian system to light, as well as the amplitude and power of the rhythm [79,80]. These differences can contribute to sex-specific susceptibilities to circadian misalignment and sleep-related problems.

## 3. Association of Disrupted Circadian Rhythms with Substance Use Disorder

Disordered substance use refers to excessive use of a drug in a way that is detrimental to self, society, or both [81]. It includes the misuse of prescription or over-the-counter drugs, illegal substances, and inhalation of chemicals for mind-altering effects. The American Psychiatric Association defines substances as drugs that have the potential to be abused, such as alcohol, cannabis, cocaine, inhalants, and prescription medications [82]. Substance use disorder has become a major health crisis affecting the well-being of many individuals globally. The negative impact of substance use disorder on physical and mental health, as well as its connection with impulsive behavior, accentuates the severity of this problem. The lifetime prevalence of substance use disorder was reported to be approximately 10% in the United States (US) [83]. In 2017, the U.S. Department of Health and Human Services declared the US opioid epidemic a public health emergency, citing an increase in opioid overdoses that resulted in over 42,000 deaths in 2016 [84]. Furthermore, according to the 2021 National Survey on Drug Use and Health (NSDUH), 57.8 million adults aged 18 and older in U.S. households experienced mental illness, whereas 44.0 million adults reported a substance use disorder in 2020 [85]. 

Substantial research indicates that the circadian system regulates reward processing, implying that substance abuse may be closely related to circadian mechanisms (Table 1) [86]. Emergency room visits for drug overdoses exhibit circadian and circannual cycles, with peak number of admissions at 18.20 h and elevated admissions around late July compared to any other time of the year [87]. Circadian irregularities can influence disordered substance use by affecting reward-related mechanisms, regardless of the sleep pathways [22,86,88]. Circadian and disordered substance use interactions can be attributed to mesolimbic neurocircuitry; circadian gene expression is significant in brain areas that underpin mood and reward functions [48]. These genes are important modulators of behavioral responses to drugs of abuse and can affect brain plasticity [25,88]. However, substance use disorder and circadian rhythms form a reciprocal relationship, as disordered substance use can impair the molecular clock function and can directly affect entrainment of the circadian system [21,89,90]. 

Furthermore, disordered substance use is closely associated with disrupted sleep, with each exacerbating the other [106]. Repeated exposure to addictive substances directly affects sleep by changing the latency, duration, and overall quality of sleep [106,107], whereas difficulties falling asleep can increase the likelihood of relapsing into substance usage [108]. All the factors discussed that disrupt circadian rhythms or sleep can play a critical role in the development of substance use disorder. Sleep issues experienced early in life can increase the chances of future involvement in substance abuse [109]. Sleep disturbances in adolescents increase the vulnerability to substance misuse by affecting cognitive functions and emotional regulation [110]. Furthermore, eveningness is consistently associated with a greater risk of anxiety and impulsive behavior, promoting alcohol intake [47]. Modifications in neural reward functions have been observed in evening chronotypes, specifically altered medial prefrontal cortex reactivity during reward anticipation, and higher ventral striatum reactivity during win outcomes [111]. In the delayed discounting test, evening chronotypes showed a propensity to favor smaller, immediate rewards over larger, delayed ones [112]. Males who displayed eveningness during late adolescence (20 years) displayed greater activation across the medial prefrontal cortex and ventral striatal in response to a notification of winning a monetary reward when tested at age 22 [113].

Research suggests a multifaceted link between chronotype, social jetlag, and substance abuse. Misalignment between biological and social timing can be commonly observed in late chronotypes that may lead to substance abuse especially increased tobacco-smoking behavior [49]. To meet social responsibilities, an increasing number of people prefer to use stimulants such as caffeine and nicotine during the day and sedatives such as hypnotics or alcohol during the night. This may result in a constant stimulant–sedation loop facilitating substance abuse [114]. Night shift workers with poor sleep quality have almost twice the risk of alcohol consumption than the day workers with good sleep [115]. Also, nightshift healthcare professionals are more susceptible to self-medicating with psychoactive substances in order to cope with sleep deprivation, work-related stress, and disturbed circadian rhythms [116]. 

Because of the intrinsically intertwined neurobiology of circadian rhythms, sleep, and substance abuse, one process can substantially influence the other. Below, we present the major substances of abuse and their likely correlation with circadian rhythms and sleep.

### 3.1. Nicotine

Cigarette smoking is a major cause underlying preventable disability and death in the US. Over 16 million Americans suffer from ailments related to smoking, and more than 480,000 individuals die due to smoking each year [117]. Today the use of electronic nicotine delivery systems, e-cigarettes (vaping), and nicotine patches has increased largely as an alternative to smoking tobacco. Several effects of nicotine can vary depending on the time of administration, and its elimination from the body also demonstrates diurnal variations [118]. Nicotine can directly affect the neurons that control the sleep–wake cycle. Cigarette smokers generally have shorter sleep time, increased daytime drowsiness, and an unusual polysomnography pattern [119]. A correlation between cigarette smoking and chronotype was reported, where smoking was significantly higher in late risers of all ages (except for those in retirement) [49]. 

Nonhuman animal studies support the human data, and a similar correlation between smoking and circadian rhythm disruption was reported for various rodent studies. In one mouse study, exposure to cigarette smoke disrupted daily rhythms of systolic blood pressure, body temperature, and heart rate measurements. Locomotor rhythms were also disrupted by exposure to cigarette smoke. These physiological and behavioral rhythms remained disrupted weeks after exposure to the smoke ended [92]. In another study, it was observed that eight weeks of passive smoking in rats altered the expression of peripheral circadian clock genes [91]. Mice exposed to environmental cigarette smoke experienced altered expression of circadian clock genes in brain and lung tissue, leading to increased lung inflammation and emphysema [93]. Alteration in reward processing also plays a vital role in smoking addiction. Mice administered nicotine chronically in drinking water displayed altered circadian rhythms in striatal dopaminergic and, to some extent, serotonergic activity [120]. Furthermore, nicotine exposure during pregnancy and lactation can have effects on circadian modulation related to memory consolidation, locomotor activity, and environmental temporal synchronization in offspring. This can be attributed to the ability of nicotine to directly influence the cholinergic system, which plays an important role in cell survival, differentiation, neurogenesis, and other critical processes [121].

### 3.2. Alcohol

Alcohol use disorder is the most prevalent SUD, costing the US economy over $250 billion annually [122]. Alcohol use has a direct impact on sleep and circadian rhythms in humans. Being a central nervous system depressant, alcohol causes rapid onset and deeper sleep during the first half of the night. However, sleep fragmentation occurs during the second half of the night, which can be attributed to sympathetic arousal caused by declining blood alcohol concentrations [123]. Sleep disturbances can be experienced even after months of stopping alcohol use. Mixed-gender individuals with alcohol use disorder demonstrate sleep disturbances irrespective of short-term or long-term abstinence [124]. 

Furthermore, alterations in circadian rhythms are present not only during alcohol use disorder and acute alcohol abstinence but also after a single acute alcohol intake. Melatonin, cortisol secretion, and core body temperature show dose-dependent changes in circadian rhythms after single acute alcohol consumption [125]. According to earlier studies on the voluntary consumption pattern of alcohol, maximum intake is observed during the active dark period in rodents [126]. When mice were exposed to ethanol over the course of several weeks either during the night or day, a substantially higher self-administration was observed later in the mice that had ethanol exposure during the night [127]. Studies suggest that the propensity to develop habitual behavior or chronobiological tolerance for alcohol is dependent on factors such as sex and genetic background [128]. Previous research has also indicated a circadian variation in the alcohol dehydrogenase activity [129]. 

It was also observed that the consumption patterns of alcohol and other substances of abuse vary at similar transitional times in which circadian chronotypes shift. Young children have the highest nocturnal melatonin surge and average melatonin levels. These levels reduce with age, declining rapidly during the first 20 years and then gradually declining until later adulthood [21]. For instance, compared to other age groups, adolescents are more likely to binge drink and use marijuana. Moreover, their preference to stay up late at night and wake up late in the morning is correlated with their degree of alcohol and marijuana usage [113,130]. Adolescents are also more likely to consume caffeine and other stimulants in order to counter daytime tiredness, which may turn into taking alcohol at night as a sleeping aid [131]. Also, older adults who have problems with their circadian and sleep cycles are more likely to abuse prescription medicines and alcohol [132]. The intricate relationship between alcohol intake and sleep/circadian rhythms highlights the need for additional research on the long-term effects of alcohol consumption.

### 3.3. Cannabis/Marijuana

There are around 193 million cannabis users around the globe [133]. According to the NSDUH (National Survey on Drug Use and Health), over 14 million persons aged 12 and older fit the criteria for cannabis use disorder in the US, with young adults aged 18 to 25 having the highest rates [134]. Multiple cannabinoid receptors have been identified in mammals, such as CB1, CB2, GPR55, and GPR18 [135]. The cannabinoid receptor (CB1R) is strongly expressed in the SCN [98]. Apart from the SCN, these receptors are also present in other brain regions which constitute a crucial part of the circadian system, such as the intergeniculate leaflet of the thalamus and both the dorsal and median raphe nuclei [99]. Anandamide (AEA or arachidonoyl ethanolamide) and 2-arachidonoyl glycerol (2-AG) are the two main endogenous cannabinoids. The endocannabinoid system regulates several components related to circadian rhythms, including body temperature, sleep, appetite, pain perception, and cognition [136,137]. The inhibition of fatty acid amide hydrolase, the enzyme inhibiting anandamide, reduces the sleep problems linked to cannabis withdrawal, supporting the sleep-promoting properties of anandamide [138]. 

The consumption of exogenous phytocannabinoids such as Δ^9^ tetrahydrocannabinol (THC) and cannabidiol (CBD) can interact with the endocannabinoid system and affect the circadian clock [139]. Sativex, an oromucosal spray containing equal parts THC and CBD, can relieve severe pain-related sleep problems possibly because of the sedative effect of THC along with the symptomatic relief from pain, spasms, and nocturia [140]. Cannabinoids can alter the ability of the SCN to entrain to light cues by modulating the circadian clock genes and influencing GABAergic transmission [98]. Long-term marijuana usage may act as an additional zeitgeber to the human circadian system [141]. When compared with a drug-free control group, chronic marijuana users abstaining during the study displayed impaired sleep efficiency with reduced slow-wave and rapid eye movement (REM) sleep in polysomnographic recordings [97]. In a study of hamsters, cannabinoid receptor agonist CP55940 suppressed light-induced phase shifts, and this action was prevented by CB1R antagonists LY320135 and AM 251 [99]. Paradoxically, there is evidence that low dosages of cannabinoids can benefit people in advanced age by entraining central and peripheral circadian clocks [136].

### 3.4. Cocaine

Cocaine is a strong sympathomimetic alkaloid derived from the leaves of *Erythroxylon coca*. It is the second most commonly used and trafficked illegal drug in the world following cannabis. The addictive potential of cocaine is high; 5% of users experience substance dependence within the first year and 20% of users develop long-term dependence [142]. It was estimated that one in every four adult, attention-deficit/hyperactivity disorder (ADHD) patients uses cocaine, and one in every ten develops a cocaine use disorder [143]. Presence of adulterants and polydrug use is often seen, with cocaine increasing the risk of toxicity [144,145]. The use of fentanyl and its analogues as adulterants in cocaine and other substances has been connected to acute intoxication and mortality [146]. Cocaine has a profound effect on circadian cycles and reward mechanisms which may play a role in the emergence and maintenance of addiction. 

Chronic administration of cocaine in rats resulted in phase reversal and blunting of circadian rhythms, by disturbing the expression of the circadian clock genes in reward-related areas [100]. Systemic administration of cocaine in mice inhibits SCN photic signaling, causing strong reduction (60%) in light-induced phase-delay shifts of circadian locomotor activity during early night. Additionally, the non-photic effects of cocaine induce circadian phase-advanced shifts (1 h) at midday. These effects can be related to the modulation of serotoninergic transmission by cocaine [101]. Cocaine can evoke tremendous pleasure and yearning due to increased mesolimbic dopamine transmission [147]. It impairs dopamine-mediated reward and plasticity mechanisms, resulting in long-term maladaptive plasticity. Cocaine’s effect on mesocortical learning pathways and its ability to induce the release of dopamine within the brain reward regions leads to abuse and dependence [144,148]. Continued use of cocaine causes desensitization, and therefore, higher doses are required to elicit stimuli of the same magnitude as before as well as to reduce the withdrawal symptoms [144]. Cocaine addiction progresses rapidly, causing serious medical, psychological, and psychosocial repercussions [14]. 

### 3.5. Opioids

Opioid-based medications are used for pain management and treatment of cough or diarrhea. However, because of their ability to induce euphoric effects, misuse of prescription drugs or illicit opioids is largely reported [149]. After alcohol and tobacco, opioid dependency is the third most prevalent substance use disorder, causing substantial morbidity and mortality [150]. The mechanisms governing opioid analgesia and opioid reward processing are modulated by the circadian clock [151]. Nonlethal overdose in people with substance use disorders exhibits a diurnal rhythm, peaking significantly in the afternoon and early evening [152]. Animal studies also support the interaction of circadian rhythms with opioid reward. For instance, in a study performed on rats, heroin self-administration was higher at night compared to the day [153]. Significant alterations are present in circadian rhythms, GABAergic and glutamatergic synaptic processes in the nucleus accumbens and dorsolateral prefrontal cortex of chronic opioid users [154].

It was not until the 1970s that the mechanism of opioids became understood with the discovery of an endogenous opioid system [155]. The endogenous opioid system has been confirmed to play an essential role in regulating reward processing, mood, motivation, learning, memory, gastrointestinal function, and pain relief [156]. Enkephalins were the first endogenous opioid peptides discovered in 1975, followed by endorphins and dynorphins [157]. 

One study confirmed that melatonin directly supports the production of enkephalins. Tissue levels of enkephalins were significantly increased in pinealectomized rats after exogenous melatonin administration or after exposure to 4 to 6 h of darkness [158]. Variations in endogenous opioid activity can predict differences in pain thresholds, opioid-induced analgesia, and the likelihood of opioid misuse in individuals [159]. Similarly, both pain and the use of exogenous opioids have the potential to interfere with the endogenous opioid system [159]. As per the National Survey on Drug Abuse 2019, a significant proportion of opioid misuse cases (>96%) were linked to prescription opioid medicines, with the primary reason being the alleviation of physical pain [156].

Fentanyl is the most potent synthetic opioid available today; it is used as an analgesic or an anesthetic clinically. Fentanyl analogs and novel non-fentanyl compounds have caused a spike in opioid-related overdose mortalities [160]. In common with all other opioids, fentanyl interferes with endogenous circadian rhythms. Fentanyl administration in mice disrupts the sleep–wake cycle and increases daytime activity displayed by prolonged free-running locomotor activity in the wheel-running behavioral test. These effects were ameliorated by pretreatment with melatonin [103]. 

Previous research has demonstrated that the sensitivity to pain and opioid-induced analgesia is influenced by circadian rhythms. A study of the effects of fentanyl analgesia in healthy volunteers revealed that the peak fentanyl analgesia effect was observed at 1730 h, whereas the lowest was around 0530 h [161]. In a study performed on post-operative patients provided with a patient-controlled opioid analgesia delivery system, the self-administration of morphine was significantly higher during the night (2300 to 0500) compared to any other time of the day [162]. 

Moreover, disruption of circadian rhythms can increase pain sensitivity. In a study carried out on rotating shift nurses, two consecutive night shifts increased sensitivity to heat pain and electrically induced pain [163]. Nurses working night shifts are also at increased risk of lower back pain, especially those who are obese [164]. Heightened pain sensitivity caused by disrupted circadian rhythms can also be a possible reason for the misuse of opioids along with other analgesic drugs. Another factor to take into consideration is opioid tolerance and opioid-induced hyperalgesia. Studies on individuals with opioid use disorder, patients, and healthy volunteers indicate that opioid administration can upsurge the pain sensitivity to certain painful stimuli [165]. Tolerance or opioid-induced hyperalgesia can be a result of modifications in μ opioid receptors, cell-to-cell interactions, or activation of an independent oppositional system [166]. The development of opioid-induced hyperalgesia might differ depending on a person’s gender, age, genetic background, immune system, level of pain, history of opiate usage, and the dosages taken [159]. Therefore, it is critical to comprehend and link the connections between endogenous opioid system, opioid reward effects, and pain management in order to combat maladaptive opioid use. 

## 4. Putative Mechanisms

### 4.1. Role of Circadian Genes and Mesolimbic Reward Pathway

Disrupted circadian rhythms are common in substance use disorders and can be related to genetic anomalies in circadian genes, leading to changes in dopaminergic pathways (Table 2) [25,167]. The initial reward phase of substance abuse is mediated by stimulation of dopamine transmission in the mesocorticolimbic circuits of the brain reward system [168]. Studies have confirmed the expression of clock genes in the brain areas associated with dopamine production, such as the ventral tegmental area and substantia nigra. Modulation of the dopaminergic pathway in the limbic system by clock genes plays a crucial role in intensifying drug-associated behavior [169]. 

#### 4.1.1. Preclinical Studies

Genetic animal models have confirmed that mutations in clock genes can produce specific phenotypes related to substance use and psychiatric disorders [167,175,179]. Multiple addiction phenotypes have been generated in animal models through knockdown/mutations of the *Per* gene [21]. One study reported that mice having clock gene mutations increased preference for the cocaine and sucrose rewards and also mimic the behavior of bipolar patients in the manic state [175]. Another study reported that mice deficient in clock genes exhibit heightened excitability of dopamine neurons in the ventral tegmental area following cocaine reward [167].

Research supports the link between alcohol use disorder and core clock gene expression [176,177]. Genetic polymorphisms in Brain and muscle ARNT-like (BMAL) 1 are related to a pattern of social drinking, whereas polymorphisms in the *Per* genes are linked with patterns of alcohol abuse [171,180]. Chronic alcohol consumption in rats leads to alternations in the diurnal core body temperature, locomotor activity, and corticosterone concentrations, along with elevated *Per1* gene expression in the adrenal and pituitary glands [173]. In another study, alcohol self-administration was considerably higher in the *Per2* mutant mice compared to the control group. This difference may be attributed to an increased glutamate level [172]. One long-term alcohol intake study in ethanol-preferring (P) and high alcohol drinking (HAD) rats demonstrated that male rats show a sex-specific escalation in ethanol preference over an extended period [181]. 

#### 4.1.2. Clinical Studies 

Researchers have explored the association between human circadian genes and substance use disorders. Clinical studies support the involvement of circadian clock genes in alcohol use disorder [178,182]. Variants of CLOCK genes were reported to be associated with the cocaine dependence in an African American population [183]. A study on Swedish adolescent boys confirmed that PER2 single nucleotide polymorphism is associated with increased alcohol consumption along with sleep-related problems [170]. The hPer1 promoter SNP rs3027172 established association with frequency of high alcohol intake in adolescents and psychological stress-related alcohol use disorder in a study performed on a German population [171]. Another study on young adults revealed that those carrying G allele for PER2 haplotype (SNP rs56013859) were less likely to drink alcohol than individuals homozygous for the A allele, thus validating the protective influence of PER2 in disordered alcohol use and stress-induced alcohol intake [184]. Furthermore, PER3 genotype significantly contributes to the intensity of insomnia symptoms in people with alcohol use disorder [185]. 

In addition to clock genes, circadian cycle-dependent dopamine receptor modifications are also linked with changes in propensity to substance abuse. Almost every aspect of dopaminergic activity, including dopamine synthesis, release, degradation, and post-synaptic effects, exhibits diurnal variation due to circadian genes Clock and Bmal1. This diurnal fluctuation can perhaps explain the difference in behavioral responses to various substances of abuse [186,187]. Research conducted on healthy individuals demonstrated that delayed phase activity rhythm (eveningness) and physical inactivity are related to enhanced dopamine receptor- D1R availability in caudate and higher D2/3R availability in nucleus accumbens, resulting in heightened sensitivity to the rewarding impact of methylphenidate [188]. An additional study verified that dopamine (D1R) and glutamate n-methyl-d-aspartate (NMDA) receptor antagonists can antagonize the effects of methamphetamine on the circadian clock [189].

### 4.2. Epigenetic Modifications

Furthermore, it has been proposed that there may be epigenetic connections between addiction and circadian rhythms. Substance abuse can be significantly correlated with histone modifications, DNA methylation, and changes in regulatory RNAs related to the circadian clock system [190]. Binge-like ethanol administration in adolescent rats increases histone (H3 and H4) acetylation, dimethylation (H3-K4) in the promoter region of cFos, Cdk5, FosB, and upregulates histone acetyl-transferase activity in the prefrontal cortex [191]. FosB is constitutively expressed in the SCN throughout the light–dark cycle [190]. Chronic cocaine exposure elevated H3 acetylation in the nucleus accumbens of rats [192]. Another study reported that a single injection of cocaine caused H4 deacetylation at the cFos promoter, whereas chronic administration showed H3 hyperacetylation at the BDNF and Cdk5 promoters in rodents [193]. Heroin administration increases H3 phosphoacetylation in the nucleus accumbens, causing enhanced heroin place preference, thus affecting reward functioning [194]. Various studies have confirmed that epigenetic modifications can be a predisposing factor as well as a response to substance abuse [195]. 

It is evident that substance use disorder involves the circadian system. However, the interrelation between substance of abuse, underlying reward circuitry, and clock genes is highly complex and specific [196]. Understanding these mechanisms can provide insight into the molecular basis of addiction.

### 4.3. Melatonin and Cortisol Rhythms

Dysregulation of endogenous melatonin and cortisol rhythms can also influence substance use disorders. Melatonin administration modulates alcohol seeking and relapse drinking behavior in male Wistar rats exposed to long-term voluntary alcohol consumption with periodic abstinence [197]. In contrast to non-pinealectomized mice, pinealectomized animals did not display any diurnal variations in the rewarding effects of cocaine in conditioned place preference tests [198]. In individuals with alcohol use disorder, acute ethanol withdrawal causes altered plasma melatonin and cortisol concentrations with loss of circadian periodicity [199]. As people age, melatonin production declines and the peak shifts to late-night hours, whereas cortisol production increases, peaking earlier in the night, exacerbating sleep difficulties [200]. Melatonin can be potentially used for the management of substance use disorders because of its anti-inflammatory, analgesic, and neuroprotective properties via influencing gut microbiota and epigenetic modifications [201].

## 5. Future Directions

Substance use disorder involves the circadian system, but the extent to which disrupted circadian rhythms can increase the vulnerability to disordered substance use or can incline an abstinent person towards relapse remains largely unspecified [202]. Circadian misalignment and sleep deprivation promote developmental tendencies toward higher reward sensitivity and impulsivity for substances such as nicotine, alcohol, and illicit drugs [86]. Disrupted sleep can also influence withdrawal in different drug users depending on the substance of abuse [203]. Current drug therapy for substance use disorder rarely addresses sleep issues, which could be a barrier to effective management [204,205]. Because of the intertwined nature of circadian rhythms, sleep, and reward function, treatment regimens focusing on sleep and circadian rhythms may be beneficial for managing substance use disorders. 

In addition, there are several variables, including chronotype, night shift work, social jetlag, and others, that can act as risk factors for disordered substance use. These factors can directly influence the development, reinforcement, and relapse of disordered substance use. Studies focusing on screening these groups for the symptoms of circadian rhythm disruption may be helpful in designing preventive interventions.

The impact of circadian rhythms on substance use disorders varies across the lifetime, from adolescence to old age [21]. Adolescents are particularly vulnerable to circadian rhythm and sleep disturbances, leading to impairment of reward function and emotional control [131]. Therefore, adolescence is the most critical period for the initiation and progression of substance use disorders. Future clinical research, particularly on adolescents and young adults, can shed light on the molecular linkages that connect one process to another. This also highlights the importance of addressing sleep and circadian disruption issues in this age group in order to prevent the development of substance use disorder in the future. Therapeutic interventions targeting the circadian system may also be effective in restoring reward-related functions by modifying dopaminergic signaling.

Another area that deserves additional study is inter-individual variation. Personalized treatment plans that consider the circadian profile, sex, and race of individuals can be extremely beneficial. Tailored treatment regimens can help develop strategies for restoring standard rhythms, reducing disordered substance use, and improving withdrawal symptoms. Future research to investigate mechanistic connections, along with the development of practical diagnostic tools to access circadian function, can be extremely beneficial. Also, meticulously designed randomized controlled clinical trials can facilitate the translation of results in clinical settings.

## 6. Conclusions

Circadian rhythm disruptions can have a profound effect on the development and progression of substance use disorders. Substance use disorders can have detrimental effects on individuals’ lives and may have serious societal implications, such as increased crime rates and health care expenses. Therefore, prevention and management of disordered substance use is essential for personal as well as societal well-being. Considering the bidirectional relationship between circadian rhythms and substance use disorders, chronotherapeutics, which include circadian clock protein modifications, melatonin agonists, light therapy, and sleep scheduling, have the potential to become adjuvant or even mainstay treatments for substance use disorders. 

## Figures and Tables

**Table 1:** Studies reporting the disruptive effects of major disordered use of substances on circadian rhythms and sleep.

Drug	Model	Major Observations	Proposed Mechanism	Reference
Nicotine	Rats (Passive smoking for 8 weeks)	Changes in circadian rhythm leading to molecular events causing IVD degeneration	Altered clock gene expression in the IVD	[91]
Nicotine	Mice (BALB/c); CS exposure (50 min twice daily for 24 weeks)	Disrupted circadian rhythms of body temperature, systolic blood pressure, heart rate, and locomotor activity	No molecular investigations reported	[92]
Nicotine	Mice (C57BL6/J and SIRT1-BMAL1mutant); CS exposure (100–300 mg/m^3^ particulates)	Reduced locomotor activity and increased lung inflammation leading to emphysema	Altered expression of the core clock gene, BMAL1	[93]
Alcohol	Rats (SD) and Mice (C57BL/6J); alcohol (3 g/kg and 5 g/kg intragastric)	Disrupted sleep homeostasis	Modulation of adenosine and effect on cholinergic neurons of the basal forebrain	[94]
Alcohol	Mice (C57BL/6J); alcohol (2%, 4%, 8% and 16%)	Adolescent alcohol intake induces circadian misalignment causing increase in adult alcohol preference	Changes in reward- and stress-related pathways	[95]
Alcohol	Humans	Reduced slow-wave sleep and increased proportions of stage 1 and REM sleep	Changes in frontal cortex structure	[96]
Marijuana	Humans	Impaired sleep efficiency with reduced slow-wave and REM sleep upon abstinence	Effect of Δ^9^-THC on CB1 receptors in the prefrontal cortex; changes in adenosine concentration	[97]
Cannabinoids	Mice (C57BL/6J); WIN55 (9 nM), AM251 (9 nM)	Attenuation in the light evoked phase shifts, altered ability of SCN to entrain to light cues	Excitatory effects in the SCN by modulating GABA release through CB1 receptor	[98]
Cannabis	Hamsters; CB(1) agonist CP55940 (0.125 mg/kg *i.p.*)	Alterations of the circadian system	Modulation of CB1 receptors in SCN, intergeniculate leaflet of the thalamus, dorsal and median raphe nuclei	[99]
Cocaine	Rats (SD); cocaine (20 mg/kg *i.p.* for 21 days)	Phase reversal and blunting of circadian rhythms	Changes in expression of clock genes in reward-related areas	[100]
Cocaine	Mice; cocaine (20 mg/kg *i.p.*)	Reduction in light-induced phase delay shifts of circadian locomotor activity	Modulation of serotonergic transmission	[101]
Cocaine	Mice (C57BL/6); cocaine hydrochloride (0.5 mg/mL)	Long lasting effects on circadian entrainment and lengthening of period of free-running circadian rhythms	Changes in SCN clock gene activity and effects on hypothalamic–pituitary axis	[102]
Fentanyl	Mice (C57BL/6); fentanyl (0.05, 0.1, and 0.2 mg/kg)	Disrupted sleep–wake cycle and increased daytime locomotor activity	Reduced expression of BMAL1 and MAO-A; increased TH	[103]
Morphine	Rats (Wistar); morphine (1 mg·kg^−1^, *i.p.*)	Phase shift effects on major physiological processes	Post-translational modification of clock proteins by activation of ERK1/2 and GSK3β in SCN, modulation in period gene expression	[104]
Morphine Sulphate, Methadone	Humans; morphine sulphate (15 mg), methadone (5 mg)	Reduced deep sleep and increased stage 2 sleep	Not suggested	[105]

**Abbreviations:** CS: Cigarette smoke; IVD: Intervertebral discs; SD: Sprague Dawley; REM: Rapid eye movement; Δ^9^ THC: Δ^9^ Tetrahydrocannabinol; SCN: Suprachiasmatic nuclei; *i.p.*: Intraperitoneal; BMAL1: Brain and Muscle Arnt-Like, TH: Tyrosine hydroxylase; MAO-A: monoamine oxidase A.

**Table 2:** Studies reporting the link between circadian gene mutations and substance use disorders.

Circadian Genes	Study Subject and Genetic Mutations	Association with Disordered Substance Use	Reference
PER2	Humans (Swedish adolescent boys); SNP 10870 (A/G)	Increased alcohol consumption and sleep problems	[170]
PER1	Mice; mPer1-mutant, Humans (adolescents); SNP rs3027172	Higher stress-related alcohol consumption	[171]
PER2	Mice; *Per2^Brdm1^* mutant	Increased alcohol intake and elevated glutamate concentration	[172]
PER1	Rats; PER1::LUC transgenic	Chronic alcohol consumption leading to higher Per1 expression in adrenal and pituitary glands	[173]
PER1/PER2	Mice; *Per1*^Brdm1^, *Per2*^Brdm1^, *Per1Per2* ^Brdm1^ mutant	Increased ethanol intake and reinforcement behavior	[174]
CLOCK	Mice; homozygous *Clock* mutant (*Clock*/*Clock*)	Increased cocaine reward and higher dopamine excitability	[167]
CLOCK	Mice; *Clock* mutant (Clk^Δ19^/Clk^Δ19^)	Increased cocaine reward and increased VTA dopaminergic activity	[175]
CLOCK	Mice; *Clock* mutant (Clk^Δ19^/Clk^Δ19^)	Increased ethanol intake and higher dopaminergic and glutamatergic activity	[176]
CLOCK	Humans; reduced expression of Clock	Chronic alcohol dependence	[177]
Circadian Clock Genes	Humans (Finnish general population)	Variants in ARNTL, ARNTL2, ADCYAP1, VIP show association with alcohol abuse and social drinking; DRD2/ANKK1 and NPY is related with alcohol dependence	[178]

**Abbreviations:** SNP: Single nucleotide polymorphism; VTA: Ventral tegmental area.

## Data Availability

Not applicable.

## Abbreviations

ADHD	Attention-deficit/hyperactivity disorder
ALAN	Artificial light at night
BMAL	Brain and muscle ARNT-like
CB1R	Cannabinoid receptor 1
CBD	Cannabidiol
Cdk5	Cyclin-dependent kinase 5
D1R	Dopamine receptor 1
ipRGCs	Intrinsically photosensitive retinal ganglion cells
NMDA	N-methyl-D-aspartate
NSDUH	National Survey on Drug Use and Health
PER	Period circadian regulator
REM	Rapid eye movement
SCN	Suprachiasmatic nuclei
SUD	Substance use disorder
THC	Δ^9^ tetrahydrocannabinol
US	United States
